# Ginsenoside compound K inhibits the proliferation, migration and invasion of Eca109 cell via VEGF-A/Pi3k/Akt pathway

**DOI:** 10.1186/s13019-022-01846-2

**Published:** 2022-05-03

**Authors:** Jianhou Huang, Dinglong Pan, Feng Liu, Yiting Hong, Gang Huang, Xiaowei Huang, Xinwen Wang, Zhiqiang Lin

**Affiliations:** 1 Department of Pharmacy, Quanzhou First Hospital Affiliated to Fujian Medical University, Quanzhou, China; 2 Department of Radiotherapy, The Second Affiliated Hospital of Fujian Medical University, Quanzhou, China; 3 Department of Orthopedics, Dongguan People’s Hospital, Southern Medical University, Dongguan, China; 4 Department of Gynaecology and Obstetrics, Quanzhou First Hospital Affiliated to Fujian Medical University, Quanzhou, China; 5 Department of Pharmacology, Quanzhou Medical College, Quanzhou, China

**Keywords:** Ginsenoside CK, Eca109, VEGF-A, Knockdown, Progression

## Abstract

**Objective:**

Esophageal cancer, one of the most common cancers in the upper digestive tract and is one of the leading cancer-related mortality worldwide. Accumulating studies found that Ginsenoside compound K (CK) has significantly anti-tumor effects, especially in the suppression of proliferation, migration, as well as invasion in various human cancers. While the effects of Ginsenoside CK in esophageal cancer have not been well studied. In our present study, we aim to explore the functions and mechanisms of Ginsenoside CK in the progression of esophageal cancer cells (Eca109).

**Methods:**

Cell Counting Kit-8 (CCK-8), wound healing, transwell and flow cytometry assays were applied to analyze the effects of Ginsenoside CK in the progression of Eca109 cell, western blot assay was used to investigate the potential downstream signaling pathway after Ginsenoside CK treatment.

**Results:**

Our study found that Ginsenoside CK can suppress cell proliferation, migration and invasion of Eca109 cell. Furthermore, the flow cytometry showed that Ginsenoside CK increased of apoptosis rates in Eca109 cell. The western blot results indicated that Ginsenoside CK decreased the expression of VEGF-A, P-Pi3k and P-Akt proteins. Moreover, the knockdown of VEGF-A gene could suppress cell proliferation, migration, invasion and induce apoptosis in Eca109 cell, and the expression of P-Pi3k and P-Akt proteins were significantly downregulated.

**Conclusions:**

Our study suggests that Ginsenoside CK inhibits the proliferation, migration, invasion, and induced apoptosis of Eca109 cell by blocking VEGF-A/Pi3k/Akt signaling pathway.

## Introduction

Esophageal carcinoma (EC) is one of the most malignant tumors worldwide, and present a great threat to the health of society [1, 2]. EC is well known by its high rate of metastasis, aggressive invasion and poor prognosis [3]. Surgery and chemotherapy are effective treatments for EC diagnosed at an early stage, while a lot of EC patients comes to recurrence or metastasis, and eventually develop to advanced stages of cancers, which present poor prognosis [4]. Therefore, it is of great urgent to explore the new and efficient treatment strategies, so as to improve the poor survival status of EC patients.

Ginsenoside CK is the main metabolic component of ginseng in human body [5]. Previous studies confirmed that Ginsenoside CK has anti-tumor, anti-inflammatory, anti-oxidation, liver protection, improving immune function and other effects [6]. Furthermore, the therapeutics values of Ginsenoside CK in tumors have well been studied in bladder cancer, colon cancer, liver cancer which can significantly suppress the proliferation ability of these tumor cells [7–9]. Vascular endothelial growth factor-A (VEGF-A), a highly specific pro-vascular endothelial cell growth factor, which can promote extracellular matrix degeneration, vascular permeability, proliferation, migration and angiogenesis of vascular endothelial cells [10, 11]. Study suggested that Ginsenoside CK inhibit tumor angiogenesis by suppressing the proliferation and migration of vascular endothelial cells, inhibiting the activity of VEGF-A and it’s signaling pathway, and the degradation of vascular extracellular matrix in neuroblastoma cells [12]. However, there are few studies focus on the effects of Ginsenoside CK on esophageal cancer cells and its molecular mechanism. As a targeted drug with high efficiency and low toxicity, Ginsenoside CK has great development potential in esophageal cancer.

In our present study, we aim to further explore the effects of Ginsenoside CK on the cell proliferation, migration, invasion, apoptosis and related mechanisms on Eca109 cell.

## Materials and methods

### Cell line and culture

Human esophageal cancer cell (Eca109) was purchased from the American Type Culture Collection (Manassas, USA). Eca109 cell maintained in RPMI 1640 medium (Gibco, USA) containing with 10% fetal bovine serum (Gibco, USA) and 1% penicillin streptomycin (Gbico, USA) in an atmosphere of 5% CO_2_ at 37 °C.

### Cell proliferation assay

The changes of cell proliferation were evaluated by employing the Cell Counting Kit-8 (CCK-8) assay. Cells (2.5 × 10^3^) per well were cultured in 96-well plate overnight. To investigate the proliferation effect of Ginsenoside CK (MedChemExpress, USA) on Eca109 cell, cells were maintained with different concentrations of Ginsenoside CK for 72 h. Similarly, lentivirus transfection cells were maintained in 96-well plate for 24 h, 48 h, 72 h or 96 h, then added with 10 μl CCK-8 reagent (New Cell & Molecular Biotech, China) to each well, and incubated for 2 h at 37 °C. The absorbance value (OD450) was detected by using the microplate reader (Bio-Tek, USA).

### VEGF-A knockdown cell line

Eca109 cells were transfected with knockdown lentivirus sh-VEGF-A, and the corresponding negative control lentivirus sh-NC which were purchased from Hanbio (Shanghai, China). Puromycin was used to screen the stably transfected cells. Western blot analysis was applied to evaluated the efficiency of lentivirus transfection.

### Wound healing assay

Eca109 cell (5 × 10^5^) were seeded in 6-well plates for 24 h and scraped by a sterile pipette tip. Cells were cultured with DMSO or Ginsenoside CK in FBS-free medium. sh-NC and sh-VEGF-A cells were maintained with FBS-free medium. The Zen Imaging software (Carl Zeiss, Germany) was applied to observe images at 0 h, 24 h and 48 h. The Image J software (USA) was applied to calculate the scratch area.

### Cell migration and invasion assays

The migration and invasion assays were detected by transwell chamber (BD, USA) with the presence of Matrigel (BD, USA) for invasion, and absence of Matrigel for migration. Cells (5 × 10^4^) per well pretreat with Ginsenoside CK or lentivirus were planted into the upper chamber with 100 μl serum-free medium, and the lower chamber with 600 μl complete medium. After incubation at 37 °C for 24 h, the lower chamber cells were fixed with 70% methanol and then stained by crystal violent (Beyotime, China). The migrated or invaded cells were counted.

### Flow cytometry

Ec109 cells (1.5 × 10^5^) were maintained in 6-well plates overnight and treated with Ginsenoside CK or DMSO for 48 h. sh-NC and sh-VEGF-A cells (1.5 × 10^5^) were planted in 6-well plates for 48 h. The rates of apoptosis cells were assessed by applying the Annexin V-FITC Apoptosis Detection Kit (Beyotime). The results were detected by the CytoFLEX flow cytometer (Beckman).

### Western blot analysis

Membrane and Cytosol Protein Extraction Kit (Beyotime, China) and Bicinchoninic Acid Protein Assay Kit (Beyotime) were used for protein extracted and quantified. Proteins were separated on 10% SDS polyacrylamide gel (Beyotime) and then blotted onto PVDF membranes (Millipore, USA). The PVDF membranes were incubated in 5% skim milk for 2 h. After that, all membranes were cultured with the primary antibodies of anti-Tubulin (1:10,000, Affinity, USA), anti-VEGF-A (1:1000, Affinity), anti-Pi3k (1:1000, Affinity), anti-P-Pi3k (1:1000, Affinity), anti-Akt (1:1000, Affinity) and anti-P-Akt (1:1000, Affinity) overnight at 4 °C. The membranes were then incubated with corresponding secondary antibody (1:3000, Affinity) for 1 h. The protein bands were finally detected by chemiluminescence detection system (ProteinSimple, USA).

### Statistical analysis

SPSS 24.0 software (SPSS Inc., Chicago, USA) was applied to data analysis, presented as mean ± SD. The difference between two groups were analyzed by Student’s t-test. P value less than 0.05 was defined as statistically significant.

## Results

### The structure of Ginsenoside CK and Eca109 cell proliferation changes after Ginsenoside CK intervention

**Fig. 1:**
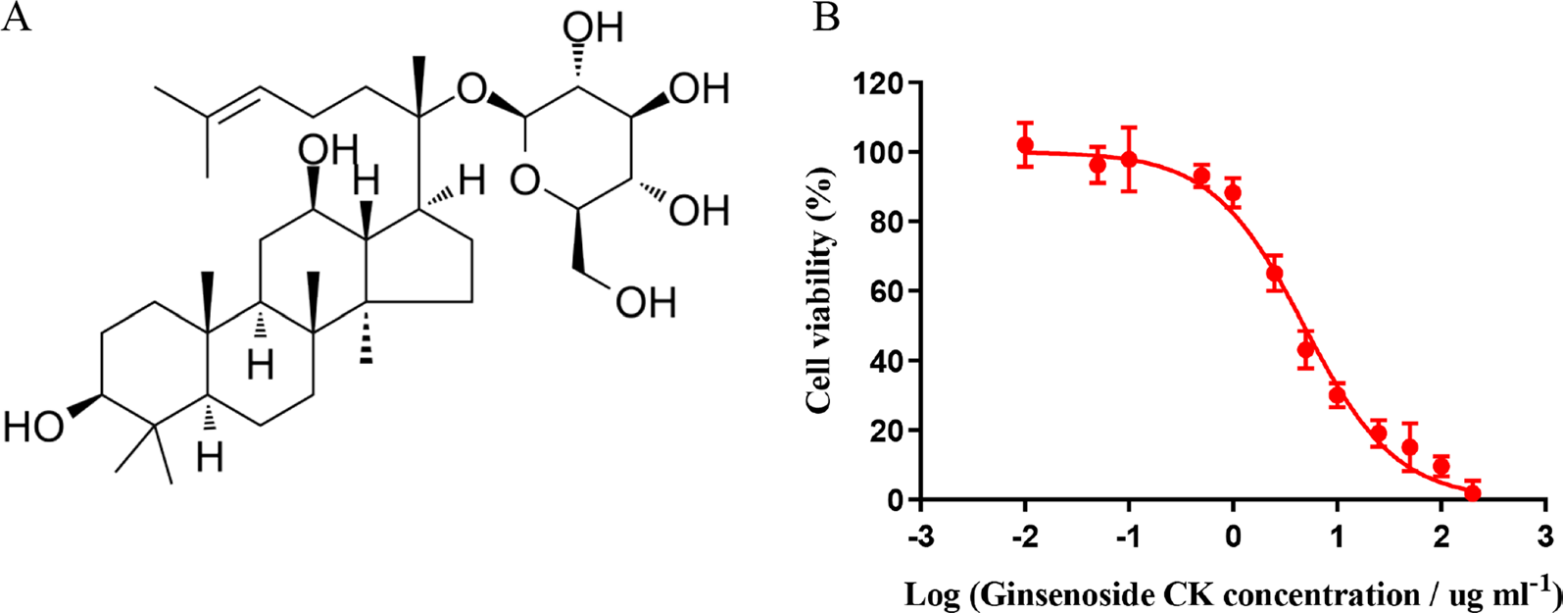
The drug formula of Ginsenoside CK (**A**) and cell proliferation changes with different concentration of Ginsenoside CK cultured for 72 h in Eca109 cell (**B**)

The structure of Ginsenoside CK was showed in Fig. 1A, CCK-8 was applied to detected the Eca109 cell proliferation changes after incubation with different concentrations of Ginsenoside CK for 72 h which found that cell proliferation was decreased with the increased concentration of Ginsenoside CK, as shown in Fig. 1B.

### Ginsenoside CK suppress cell migration and invasion of Eca109

**Fig. 2:**
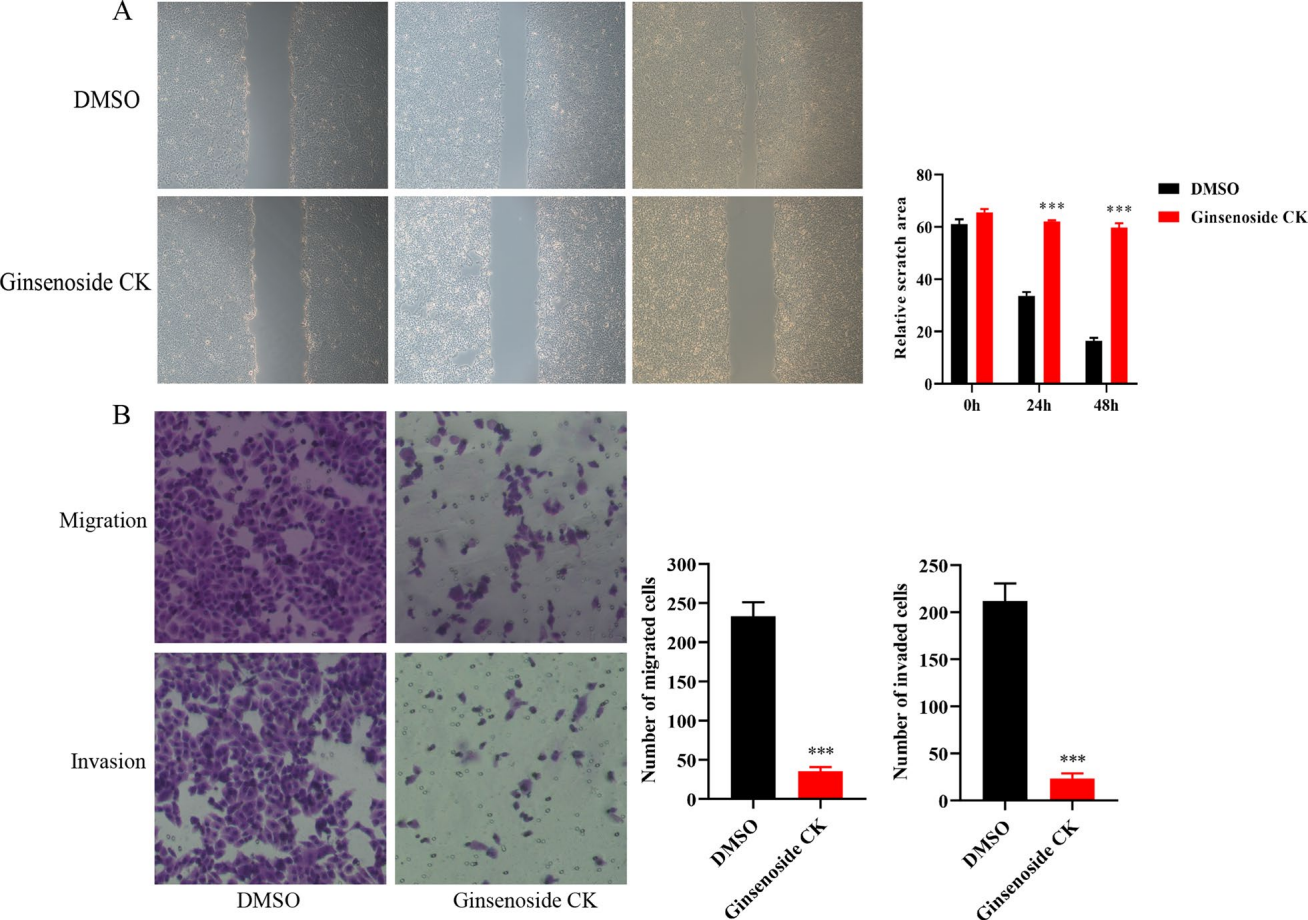
Ginsenoside CK suppressed the migration and invasion of Eca109 cell. **A** Wound healing assay showed that the migration ability was reduced after Ginsenoside CK treatment. **B** Transwell assay demonstrated that cell migration and invasion ability decreased after Ginsenoside CK intervention. ***P < 0.001

Wound healing and transwell assays were used to investigated the migration and invasion abilities after Ginsenoside CK intervention, and the results indicated that the migration and invasion abilities were reduced notably in Eca109 cell after the treatment of Ginsenoside CK (Fig. 2A, B).

### Knockdown of VEGF-A gene suppress cell proliferation, migration and invasion of Eca109

**Fig. 3:**
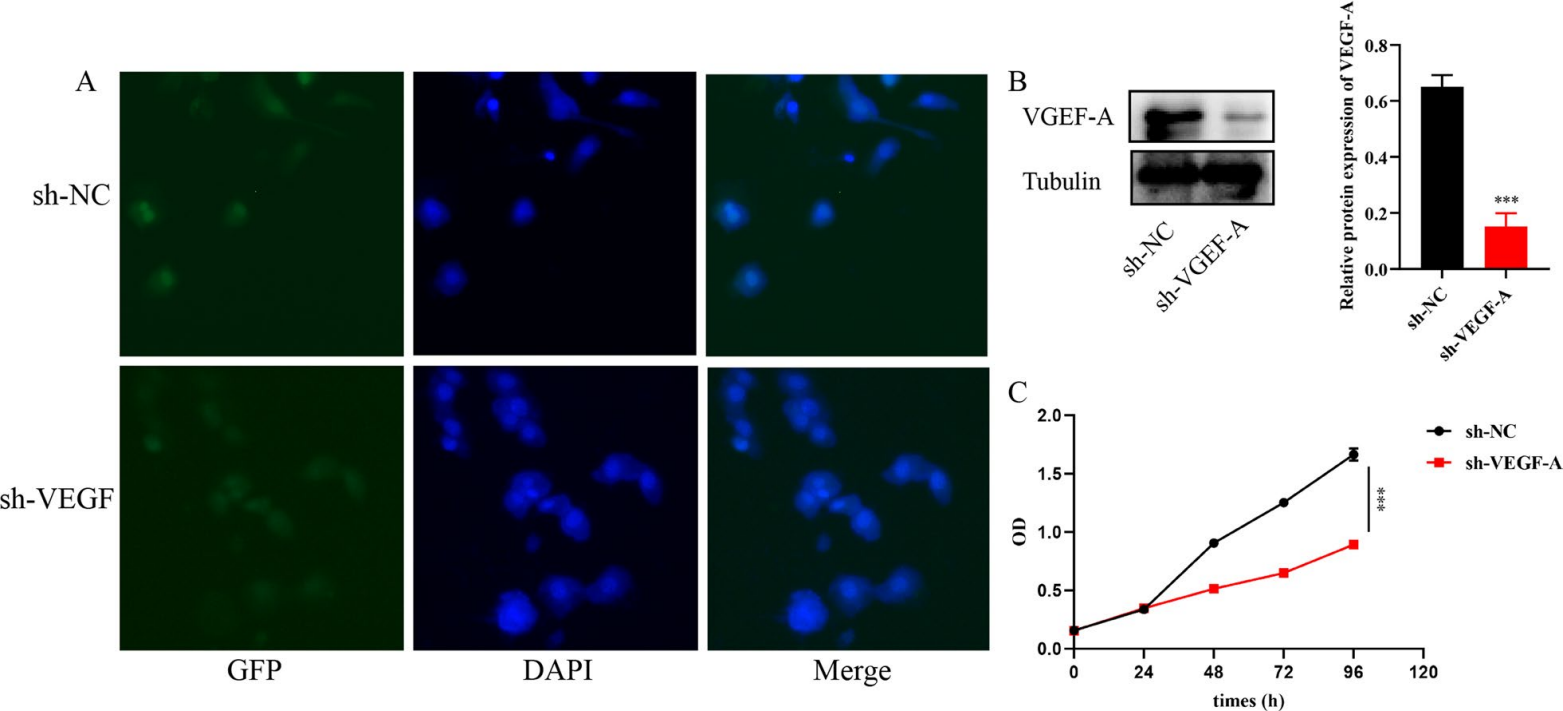
The knockdown of VEGF-A in Eca109 cell and cell proliferation changes. **A** Green fluorescent and western bolt analysis showed that Eca109 cell were stably transfected with VEGF-A knockdown gene. **B** Cell proliferation changes detected by CCK-8 assay showed that cell proliferation decreased after incubation with Ginsenoside CK for 24, 48, 72 and 96 h. ***P < 0.001

**Fig. 4:**
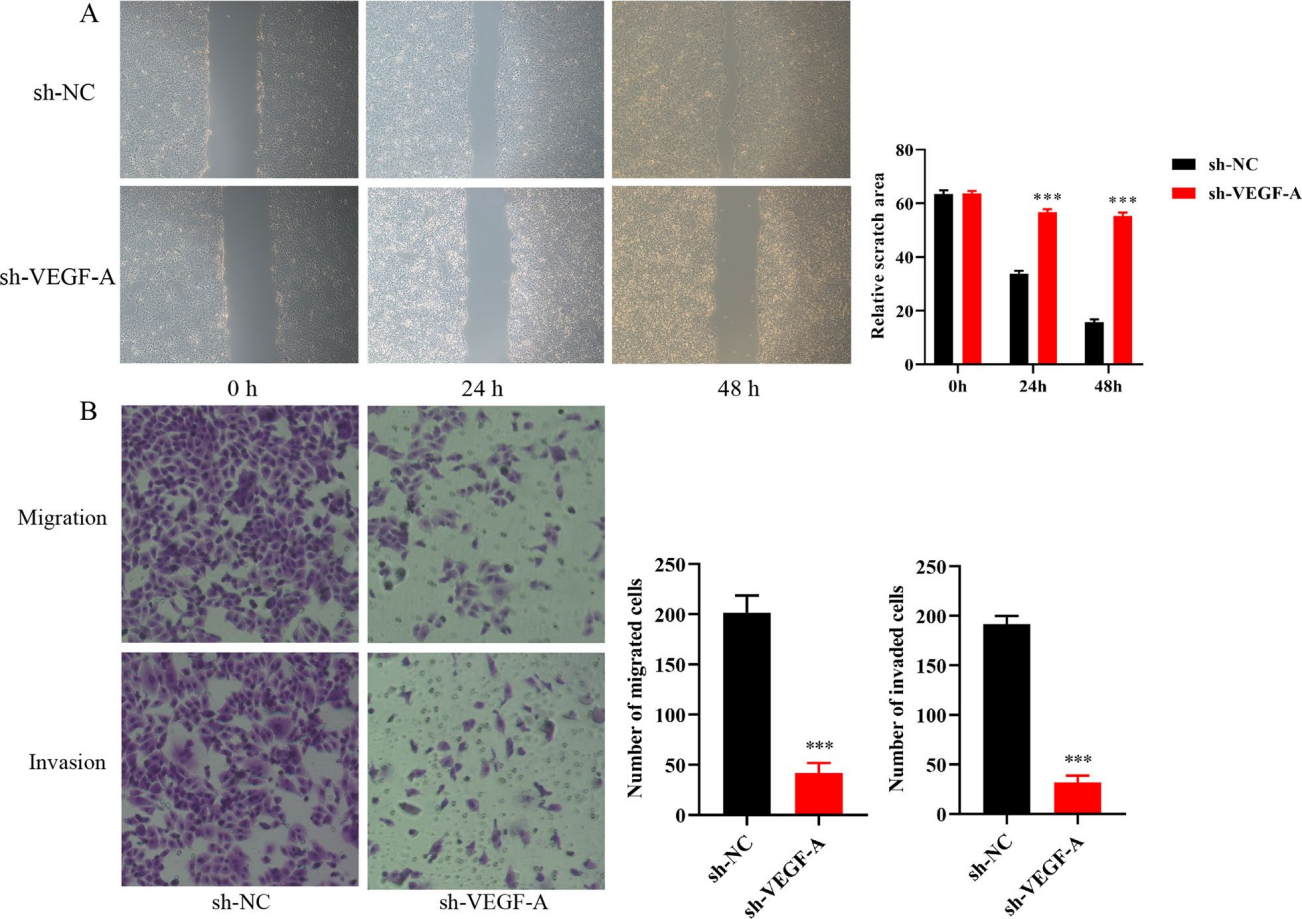
VEGF-A gene knockdown suppressed the migration and invasion of Eca109 cell. **A** Wound healing assay found that cell migration ability was inhibited after VEGF-A gene knockdown. **B** The migration and invasion ability suppress in VEGF-A gene knockdown group. ***P < 0.001

Eca109 cell were transfected with VEGF-A knockdown lentivirus (sh-VEGF-A) or the corresponding negative control lentivirus (sh-NC) (Fig. 3A). The western blot analysis confirmed that Eca109 cell were stably transfected with lentivirus (Fig. 3B). The CCK-8 assay showed that cell proliferation was remarkably reduced after sh-VEGF-A transfection (Fig. 3C). Wound healing and transwell assays further confirmed that VEGF-A gene knockdown suppress the proliferation, migration and invasion of Eca109 cell (Fig. 4A, B).

### Ginsenoside CK intervention and knockdown of VEGF-A gene promote cell apoptosis in Eca109 cell

**Fig. 5:**
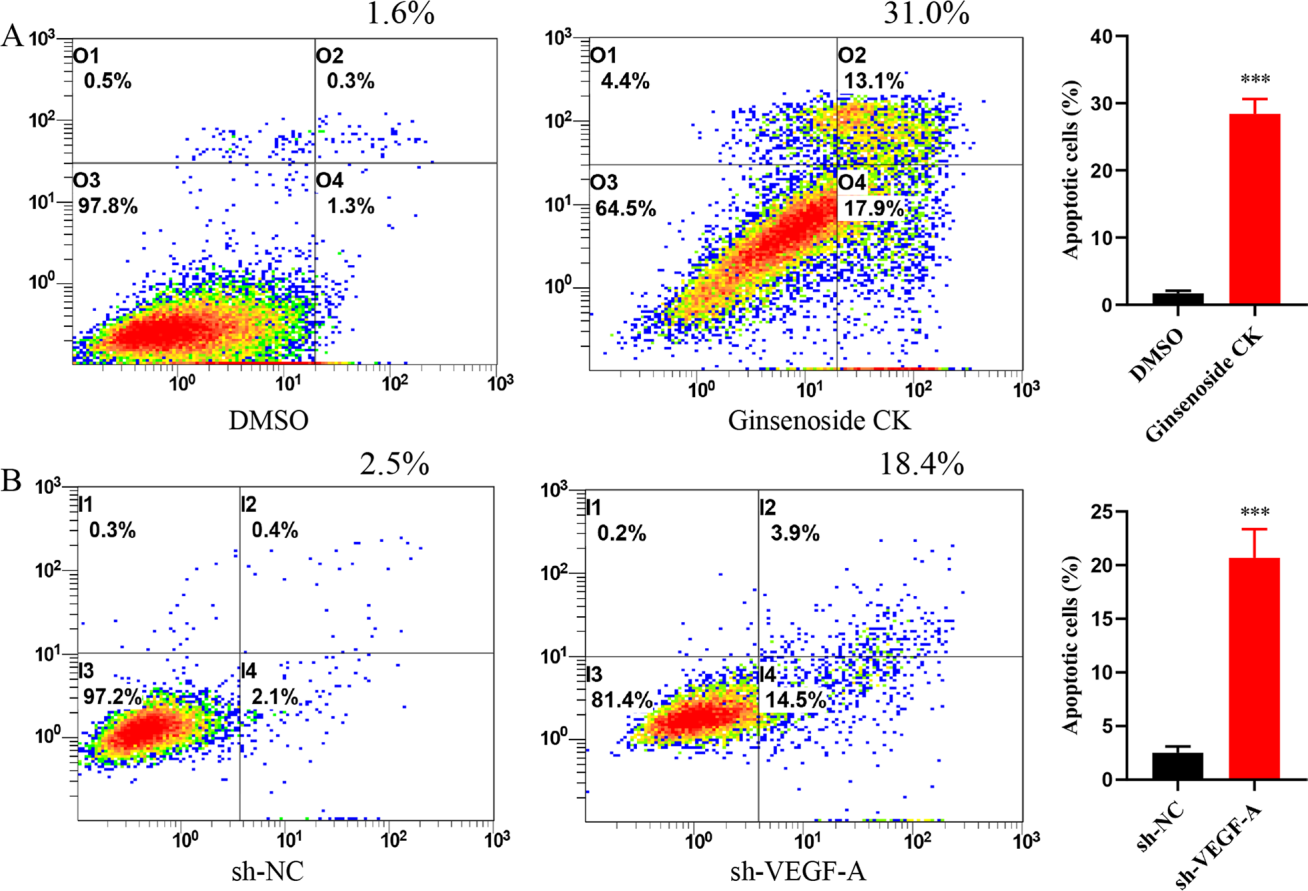
The involvement of Ginsenoside CK and VEGF-A in the pro-apoptotic in Eca109 cell. **A** The cell apoptosis rate of Eca109 cell was increased after Ginsenoside CK treatments. **B** The VEGF-A gene knockdown promote the cell apoptosis rates of Eca109 cell. ***P < 0.001

To investigate the function of Ginsenoside CK intervention and VEGF-A gene knockdown in cell apoptosis, Annexin V-FITC and PI staining was applied. The flow cytometry analysis of apoptosis showed that Ginsenoside CK intervention and VEGF-A gene knockdown promoted the apoptosis rate of Eca109 cell (Fig. 5).

### Ginsenoside CK influences Eca109 cell progression via VEGF-A/Pi3k/Akt pathway

**Fig. 6:**
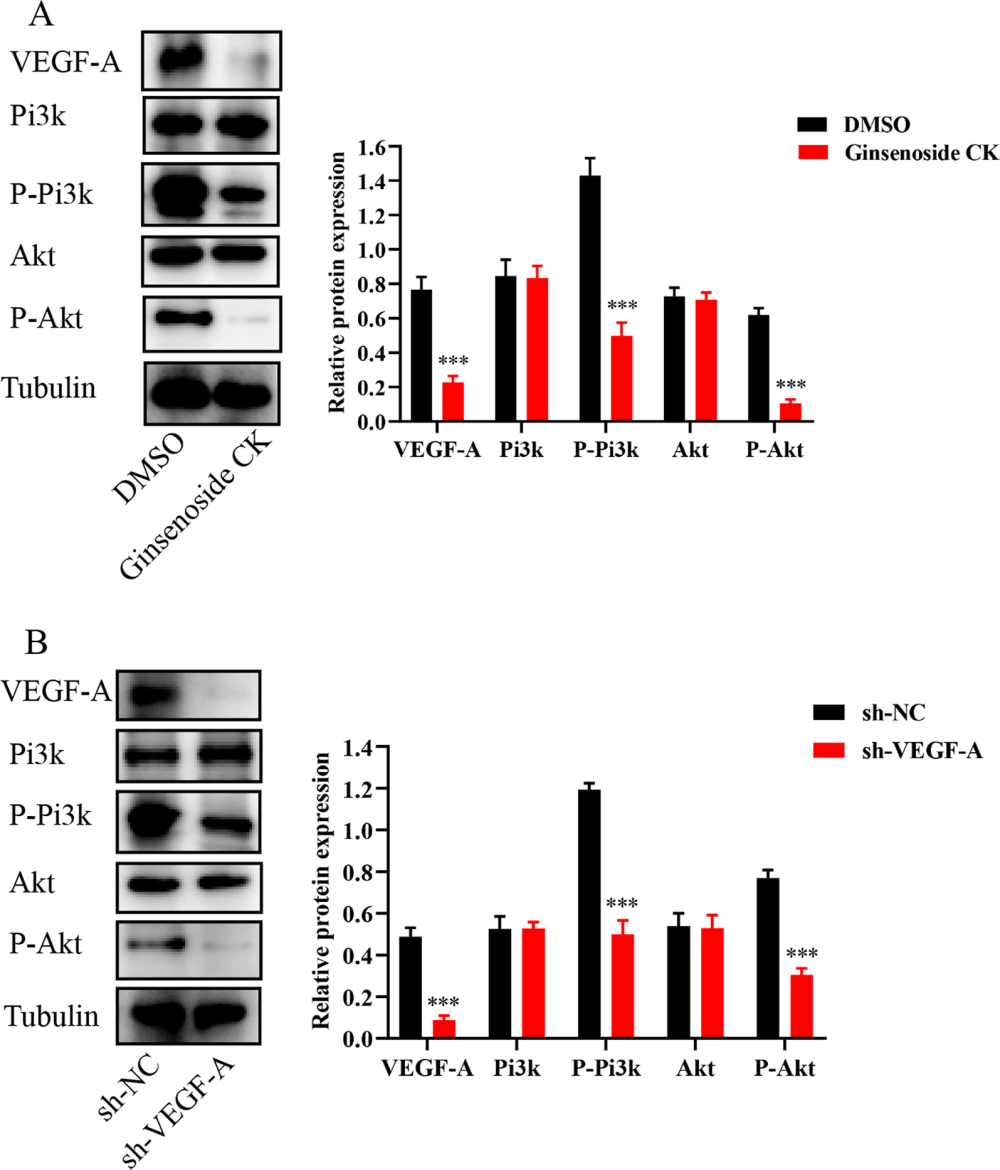
The involvement of VEGF-A/Pi3k/Akt pathway in Ginsenoside CK intervention and VEGF-A gene knockdown of Eca109 cell. The decreased expressions of VEGF-A, P-Pi3k, P-Akt in Ginsenoside CK intervention (**A**) and VEGF-A gene knockdown (**B**) groups, while the total proteins of Pi3k and Akt were unchanged. ***P < 0.001

It is confirmed that the VEGF-A/ Phosphoinositide 3-kinase (Pi3k)/protein kinase B (Akt) signaling pathway play important roles for the tumor progression on proliferation, migration, and invasion behaviors [13]. The present study detected the decrease expression of VEGF-A, P-Pi3k, and P-Akt proteins after Ginsenoside CK intervention, while the total proteins of Pi3k and Akt were unchanged. The knockdown of VEGF-A inhibits the cell proliferation, migration, invasion and induce apoptosis in Eca109 cell. Moreover, the knockdown of VEGF-A gene lead to the decrease expression of P-Pi3k, and P-Akt proteins (Fig. 6), which suggested that the VEGF-A/Pi3k/Akt pathway may present as downstream of Ginsenoside CK treatment in Eca109 cell.

## Discussion

Esophageal cancer is a highly malignant digestive tract tumor, ranking the 5th among the causes of cancer-related death in worldwide [14]. Although the diagnostic techniques and treatment methods for esophageal cancer have been continuously improved recent decade, the overall survival status of esophageal cancer patients is still unsatisfactory due to the characteristics of early invasion and distant metastasis [15]. Ginseng, as a treasure of human pharmaceutical culture, contains a variety of active components, among which ginsenoside is the main bioactive compound in ginseng and has broad implications in human disease [16]. Ginsenoside CK is a natural diol-type ginsenoside with medicinal activity in vivo [17]. Studies found that Ginsenoside CK has inhibiting effects on various tumor cells, containing lung cancer, colon cancer, and liver cancer, as well as bone tumor [18–20]. The anti-tumor effects of Ginsenoside CK are mainly reflected in its ability to reduce the proliferation, migration, and invasion in tumor cells [21]. Chen et al. [22] found that Ginsenoside CK can induce cell apoptosis and inhibit the biological activities of human osteosarcoma cells via blocking the Pi3k signaling pathway. Oh et al. [23]revealed that Ginsenoside CK promote cell autophagic and apoptosis which can inhibit human neuroblastoma cells viability both in vitro and vivo. In the present study, we investigated the anti-tumor effects of Ginsenoside CK and its related mechanism in Eca109 cell, which found that Ginsenoside CK can suppress Eca109 cell proliferation, migration, invasion, induce apoptosis and down-regulated the expression of VEGF-A, P-Pi3k, and P-Akt proteins after Ginsenoside CK intervention.

VEGF-A playing as an important regulator of angiogenesis and presenting as the mediator of endothelial cells proliferation [24]. Down-regulating the expression of VEGF-A can suppress tumor progression in gastric cancer [25]. Zhang et al. [26] reported that the blockage of VEGF-A can inhibits the proliferation and invasion of breast cancer cells. Pi3k/Akt signaling pathway can be activated by angiogenesis inducers and growth factors, like angiopoietins and VEGF-A [27]. Some studies found that Pi3k/Akt presented as the main downstream signaling pathway mediating the biological effects of VEGF-A, and the VEGF-A/Pi3k/Akt signaling pathway playing significant roles in proliferation, migration and invasion of various cellular processes [28]. Study confirmed that VEGF-A/Pi3k/Akt signaling pathway present an important role in the proliferation, migration and invasion of renal carcinoma cells [29]. In our study, we observed that Ginsenoside CK intervention decrease the expression of VEGF-A, P-Pi3k, and P-Akt proteins. Therefore, we speculated that the VEGF-A/Pi3k/Akt signaling pathway may present as the downstream in Eca109 cell after Ginsenoside CK intervention. Furthermore, the VEGF-A gene knockdown investigation showed VEGF-A down-regulation inhibit the cell proliferation, migration, invasion, induce apoptosis and decrease expression of P-Pi3k, and P-Akt proteins in Eca109 cell which further supported the hypothesis that Ginsenoside CK suppress the progression of Eca109 cell via affecting VEGF-A/Pi3k/Akt signaling pathway.

## Conclusions

In conclusion, our present study found that Ginsenoside CK can suppress the cell proliferation, migration, invasion, and induce apoptosis of Eca109 cell via VEGF-A/Pi3k/Akt signaling pathway, which suggests that Ginsenoside CK may serve as an effective treatment in EC.

## Data Availability

The data the support the findings of this study are available on request from corresponding author.

## Abbreviations

EC: Esophageal carcinoma
CK: Compound K
VEGF-A: Vascular endothelial growth factor-A
CCK-8: Cell Counting Kit-8
Pi3k: Phosphoinositide 3-kinase
P-Pi3k: Phosphorylated-phosphoinositide 3-kinase
Akt: Protein kinase B
P-Akt: Phosphorylated-protein kinase B
